# Radiocesium-bearing microparticles cause a large variation in ^137^Cs activity concentration in the aquatic insect *Stenopsyche marmorata* (Tricoptera: Stenopsychidae) in the Ota River, Fukushima, Japan

**DOI:** 10.1371/journal.pone.0268629

**Published:** 2022-05-20

**Authors:** Yumiko Ishii, Hikaru Miura, Jaeick Jo, Hideki Tsuji, Rie Saito, Kazuma Koarai, Hiroki Hagiwara, Tadayuki Urushidate, Tatsuhiro Nishikiori, Toshihiro Wada, Seiji Hayashi, Yoshio Takahashi

**Affiliations:** 1 Environmental Impact Assessment Section, Fukushima Branch, National Institute for Environmental Studies, Tamura, Fukushima, Japan; 2 Meteorology and Fluid Science Division, Sustainable System Research Laboratory, Central Research Institute of Electric Power Industry, Chiba, Japan; 3 Fukushima Prefectural Centre for Environmental Creation, Tamura, Fukushima, Japan; 4 Japan Atomic Energy Agency, Sector of Fukushima Research and Development, Fukushima, Japan; 5 Agricultural Radiation Research Center, Tohoku Agricultural Research Center, National Agriculture and Food Research Organization, Fukushima-shi, Fukushima, Japan; 6 Institute of Environmental Radioactivity, Fukushima University, Fukushima, Japan; 7 Department of Earth and Planetary Science, Graduate School of Science, The University of Tokyo, Bunkyo-ku, Tokyo, Japan; Northwestern University Feinberg School of Medicine, UNITED STATES

## Abstract

After the Tokyo Electric Power Company Fukushima Daiichi Nuclear Power Plant accident in Japan, freshwater ecosystems near the site remained contaminated by radiocesium (RCs). Clarifying RCs concentrations in aquatic insects is crucial because fishes consume these insects that transfer RCs into freshwater ecosystems. As aquatic insects are usually measured for radioactivity in bulk samples of several tens of insects, variation in RCs concentration among individuals is not captured. In this study, we investigated the variability in ^137^Cs activity concentration in individual aquatic insects in detritivorous caddisfly (*Stenopsyche marmorata*) and carnivorous dobsonfly (*Protohermes grandis*) larvae from the Ota River, Fukushima. Caddisfly larvae showed sporadically higher radioactivity in 4 of the 46 caddisfly larvae, whereas no such outliers were observed in 45 dobsonfly larvae. Autoradiography and scanning electron microscopy analyses confirmed that these caddisfly larvae samples contained radiocesium-bearing microparticles (CsMPs), which are insoluble Cs-bearing silicate glass particles. CsMPs were also found in potential food sources of caddisfly larvae, such as periphyton and drifting particulate organic matter, indicating that larvae may ingest CsMPs along with food particles of similar size. Although CsMP distribution and uptake by organisms in freshwater ecosystems is relatively unknown, our study demonstrates that CsMPs can be taken up by aquatic insects.

## Introduction

Since the Fukushima Daiichi Nuclear Power Plant (FDNPP) accident in March 2011, radiocesium (RCs; ^134^Cs and ^137^Cs) has become a major radionuclide contaminant in many areas of eastern Japan. Contaminated local freshwater fish remains a serious concern because freshwater fish accumulate higher levels of RCs than do marine fish [1]. Because aquatic insects are essential for RCs transfer into freshwater ecosystems and in fishes, understanding the distribution of RCs in aquatic insects and their RCs uptake sources in aquatic environments is important. Aquatic insects are usually measured for radioactivity in bulk samples of several tens of insects. It is labor-intensive to collect sufficient quantities of the same species, and becomes harder for small or uncommon species. Although RCs activity measurement using either one or several individual insects may be useful, investigating the variation in RCs concentration between individuals is necessary to ensure that small sample groups are truly representative of RCs concentration in them.

In the present study, we investigated the variability in ^137^Cs activity concentration of two aquatic insect larvae, *Stenopsyche marmorata* and *Protohermes grandis*. Both species are relatively large aquatic insects: *S*. *marmorata* and *P*. *grandis* are approximately 30–40 mm long and 60–70 mm long, respectively, and have different feeding habits. The larvae of *S*. *marmorata* (Net-spinning Caddisfly) feed on drifting organic matter, including diatoms, periphyton, and detritus [2] and those of *P*. *grandis* (Dobsonfly) are carnivorous predators of other aquatic insects [3]. RCs concentrations in aquatic insects are different depending on their feeding habits, and detritivores have showed to contain higher RCs concentrations than those in carnivores [4]. The high ^137^Cs activity concentration in detritivorous caddisfly larvae has been affected by ^137^Cs in their gut contents, including clay minerals [5]. As caddisflies feed on drifting particulates that are similar in size to radiocesium-bearing microparticles (CsMPs), we hypothesized that drifting CsMPs in river water consumed by caddisfly larvae could be included in their gut content and be responsible for the measured ^137^Cs activity concentration.

CsMPs are insoluble, Cs-bearing silicate glass particles. Although RCs is released into the atmosphere as water-soluble sulfate aerosol particles [6, 7], some RCs is emitted as insoluble CsMPs. The total RCs deposited on land is 3–6 PBq [8], but the ratio of RCs abundance in CsMPs to water-soluble RCs is unknown. CsMPs are important for understanding and predicting the fate of RCs in the environment and their uptake by organisms [9, 10]. After CsMPs were first reported by Adachi et al. [11], subsequent studies revealed two types of CsMPs: Type A and Type B particles [12]. The matrix of the Type-A particles is silicon dioxide (SiO_2_) glass with Cs and various other elements [13, 14]. The diameter of the Type-A particles is approximately 0.1–10 μm and their ^137^Cs activity is ~10^−2^–10^2^ Bq/particle. Type-A particles have been concluded to be emitted from Units 2 or 3 of the FDNPP because isotopic ratios of uranium [15] and Cs [15, 16] agreed with those theoretically calculated from the burnup of nuclear fuel [17]. Type-B particles were emitted from Unit 1 of the FDNPP [18]. The matrix of Type-B particles is also SiO_2_; however, they are larger (~50–400 μm) and have ~10,000 fold lower concentration (Bq/mm^3^) of ^137^Cs than those of Type-A particles [16].

Type-A particles have been found in suspended solids (SS) in the water of the Kuchibuto River in Fukushima Prefecture [18]. Miura et al. [10] also reported Type-A particles in various marine samples, such as SS and plankton net samples, which were probably transported from rivers. Therefore, CsMPs in aquatic environments may be taken up by aquatic organisms, such as aquatic insects and fish, and the radiological risk of CsMPs to aquatic organisms because of their intense radioactivity is concerning. However, the distribution of CsMPs and their uptake by organisms in aquatic systems have remained poorly investigated.

Therefore, we aimed to investigate the presence of CsMPs in aquatic insects and their food sources in the Ota River in Fukushima, Japan. Food sources of aquatic insects such as caddisfly larvae, include periphyton, SS, and coarse particulate organic matter (CPOM). CPOM are organic materials larger than 1 mm derived from plants, dead insects, and frass. We determined the distribution of CsMPs in the river environment by comparing the frequency of CsMPs found in aquatic insects and their food sources, and realized the effect of CsMPs on the variability of ^137^Cs activity concentration in these samples.

## Materials and methods

### Field sites

**Fig 1:**
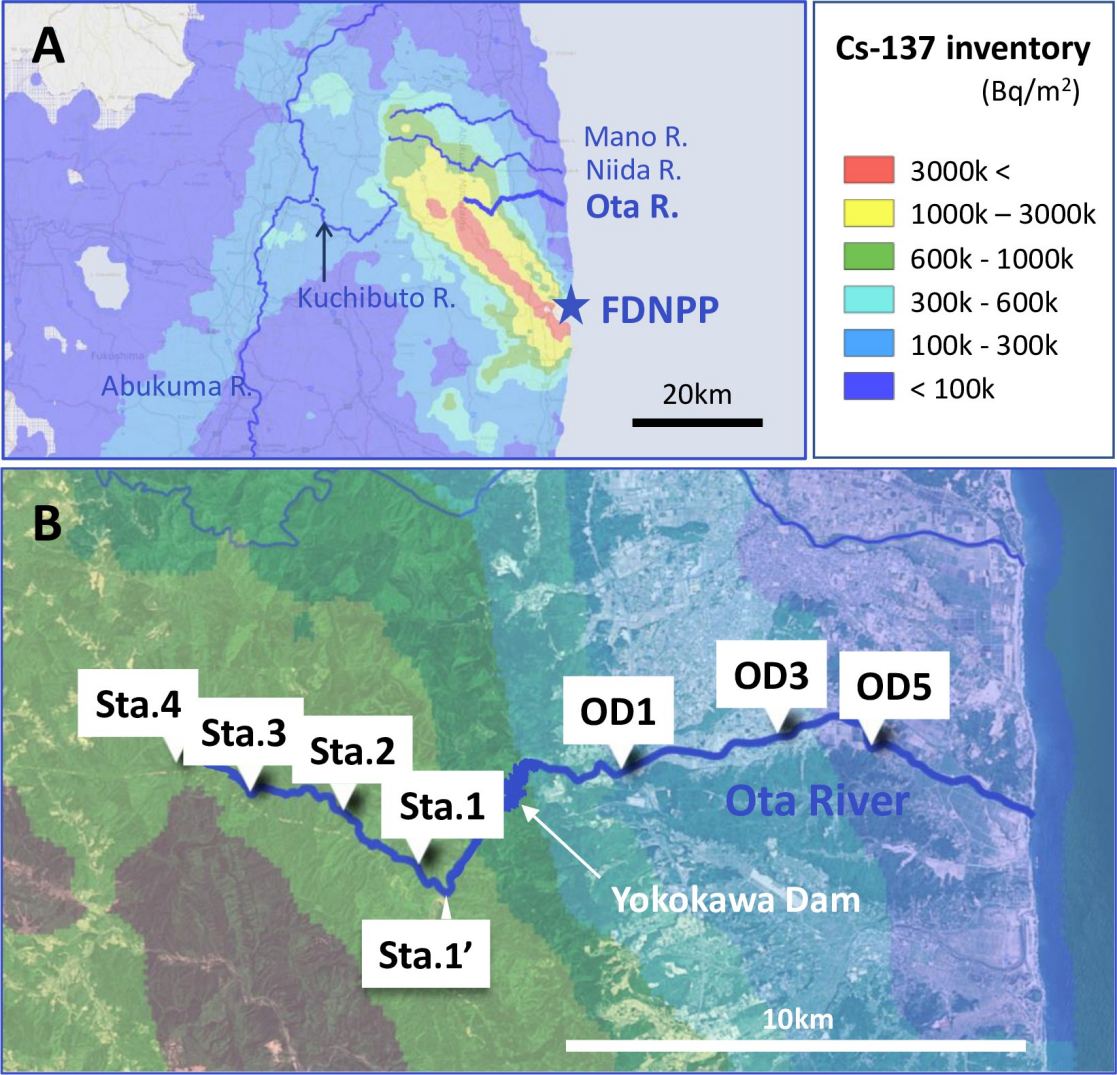
Map of the sampling sites at Ota River in Fukushima. The location of the Ota River and the Fukushima Daiichi Nuclear Power Plant (FDNPP) in Fukushima Prefecture (A) and a close-up of the sampling sites (B) are shown. The initial ^137^Cs inventory data are derived from the study of Kato and Onda [30]. The map is based on the map obtained from Geospatial Information Authority of Japan under a CC BY license.

The study was conducted on the Ota River, which is approximately 20 km north of the FDNPP and runs through the coastal area of Fukushima Prefecture (Fig 1A). The study sites were four sites from station (Sta.) 1–4, with Sta. 1 being in the forested area upstream of the Yokokawa Dam, and three sites, OD1, OD3, and OD5, in urban areas downstream of the dam (Fig 1B). The ^137^Cs inventory was higher in the upstream area (Fig 1B; Table 1).

**Table 1:** Information of the sampling sites along Ota River.

Sampling site	Latitude, longitude	^137^Cs inventory (kBq/m^2^)	Elevation (m)	Aquatic insect sampling date	Periphyton sampling date	CPOM sampling date	SS sampling date
**Sta.4**	37.59882 N, 140.81549 E	2280	446	–	3 Aug, 4 Oct, 5 Dec	3 Aug, 5 Oct, 4 Dec	–
**Sta.3**	37.59314 N, 140.83373 E	2274	380	3 Aug, 4 Dec	3 Aug, 4 Oct, 5 Dec	3 Aug, 5 Oct, 4 Dec	–
**Sta.2**	37.58950 N, 140.85667 E	1632	303	3 Aug, 4 Oct, 4 Dec	3 Aug, 4 Oct, 5 Dec	3 Aug, 5 Oct, 4 Dec	–
**Sta.1**	37.57897 N, 140.87520 E	1169	194	3 Aug, 4 Dec	3 Aug, 4 Oct, 5 Dec	3 Aug, 5 Oct, 4 Dec	–
**Sta.1’**	37.57463 N, 140.88126 E	1159	164	–	–	–	8 July, 19 August, 14 Oct (2014)
**OD1**	37.59921 N, 140.92852 E	252	43	29 Aug, 24 Oct, 5 Dec	29 Aug, 24 Oct, 5 Dec	–	–
**OD3**	37.60431 N, 140.96360 E	129	20	–	29 Aug, 24 Oct, 5 Dec	–	–
**OD5**	37.60272 N, 140.98739 E	78	7	29 August	29 Aug, 24 Oct, 4 Dec	–	–

Aquatic insects, periphyton, and coarse particulate organic matter (CPOM) samples were collected in 2018. Suspended solids (SS) samples were collected 2014. ^137^Cs inventory were calculated as the mean value in a radius of 1 km of each study site using the data of the reconstructed initial fallout map of the Fukushima accident derived radiocesium (RCs) based on analysis of the airborne monitoring survey dataset [30].

### Sampling and sample treatment

Aquatic insects were collected from the Ota River between August and December 2018. *S*. *marmorata* and *P*. *grandis* are common species, and no special permission was required for collection of these aquatic insects in this area. Larvae of caddisflies and dobsonflies were collected at Sta.1, Sta.2, Sta.3, OD1, and OD5 (only caddisfly larvae were collected at OD5; Table 1). The aquatic insects were immediately preserved in 70% ethanol after sampling. After larval samples were dried at 60°C for approximately 48 h, their whole-body individual dry weights were measured (XSE205 DualRange; Mettler Toledo Ltd., Tokyo, Japan). Each dried individual larva was ground and homogenized. Radioactivity measurements of samples collected at OD1 were conducted for each individual, but 9 out of 46 caddisfly samples were measured by lumping two to four small individuals together. For caddisfly and dobsonfly samples collected at Sta.1, Sta.2, Sta.3, and OD5, ^137^Cs was measured in bulk samples of 3–16 individuals.

Periphyton and CPOM samples were collected from August to December 2018, at the same site and date as the aquatic insects. Periphyton samples were gathered by washing off the riverbed rock surface and rinsing with distilled water, which was returned to the laboratory and centrifuged at 11,000 ×g at 10°C for 30 min. The supernatant was discarded and periphyton was dried at 60°C for approximately 48 h. After the dry weights of periphyton samples were determined, they were ground and homogenized in a mill (IFM-800DG; Iwatani Ltd., Tokyo, Japan). CPOM was collected using a draft net (1,000 μm mesh size, net opening: 25 cm×25 cm) for 1–4 h, which were wet-sieved using a 1,000 μm mesh in the laboratory, and the remainder of the sieve (> 1,000 μm) was dried at 60°C for 48 h. After dry weights were determined, samples were ground and homogenized in a mill.

The SS samples were collected in 2014 at Sta. 1’. SS in the river water was monitored once or twice a month by filtering 18–38 L of water through a 1 μm mesh polypropylene cartridge filter (Japan Vilene Ltd., Tokyo, Japan) [19]. The SS samples were collected under both base flow conditions (samples J and K, see Table 2) and runoff conditions (sample L, see Table 2). After wet cartridges were measured for ^137^Cs activity using high-purity germanium coaxial detectors, as described below, these were dried for 48 h at 105°C. The weight of the SS collected by the cartridge was calculated from the weight difference of the cartridge before water filtering and after drying. Cartridges were then dismantled by hammering, taking care not to dissipate the SS on the filter; only the filter section was extracted for CsMP analysis.

**Table 2:** Information of the samples used for autoradiography and the radiocesium-bearing microparticles (CsMPs).

	Bulk							CsMPs			Radioactivity fraction of CsMPs (%)^c^	Frequency of CsMPs (per gram)^d^	Sample particle size (μm)
	ID	Sampling site	Sampling date	Sample weight (g)	Total ^137^Cs activity (Bq)	^137^Cs activity concentration with CsMPs (Bq/g)	^137^Cs activity concentration without CsMPs (Bq/g)^a^	Total Number of CsMPs	^137^Cs activity (Bq)^b^	Total ^137^Cs activity (Bq)			
**Caddisfly larvae**	**A (OTD8-m2)**	OD1	29 Aug, 2018	0.13	1.89 ± 0.03	14.6 ± 0.2	1.07 ± 0.03	1	**1.82 ± 0.02**	1.82	92.9	5.3–15.9	Gut content: FPOM 0.45–1000
	**B (OTD7-1)**	OD1	29 Aug, 2018	0.11	0.85 ± 0.01	7.7 ± 0.1	0.39 ± 0.03	1	0.90	0.90	95.4		
	**C (OTD11-4)**	OD1	24 Oct, 2018	0.02	0.80 ± 0.02	39.8 ± 0.8	1.22 ± 0.07	1	0.74	0.74	96.8		
	**D (OTD11-2)**	OD1	24 Oct, 2018	0.05	0.13 ± 0.003	2.2 ± 0.05	0.51 ± 0.05	1	0.10	0.10	77.6		
**Periphyton **	**E (OTU84)**	Sta.2	4 Oct, 2018	8.07	227.20 ± 2.4	28.2 ± 0.3	27.3 ± 0.3	7	**2.46 ± 0.02,** 0.71, 0.69, 0.24, 0.20, 0.18, 0.16	4.65	2.0	0.1–0.8	All particle sizes
	**F (KDB20)**	OD3	24 Oct, 2018	1.82	20.30 ± 0.18	11.2 ± 0.1	9.3 ± 0.1	1	**3.39 ± 0.03**	3.39	16.7		
**CPOM**	**G (OTU153)**	Sta.1	5 Oct, 2018	4.50	50.9 ± 1.3	11.3 ± 0.3	10.4 ± 0.3	2	**2.31 ± 0.02**, 0.55	2.86	5.6	0.3–0.5	> 1000
	**H (OTU155)**	Sta.2	5 Oct, 2018	8.12	105.1 ± 1.6	12.9 ± 0.2	12.7± 0.2	9	0.69, 0.39, 0.25, 0.20, 0.18, 0.18, 0.18, 0.13, 0.13	2.34	2.2		
	**I (OTU159)**	Sta.4	5 Oct, 2018	23.72	286.4 ± 4.7	12.1 ± 0.2	12.0 ± 0.2	8	0.28, 0.23, 0.20, 0.18, 0.17, 0.16, 0.15, 0.10	1.63	0.6		
**SS**	**J (SS312)**	Sta.1’	8 July, 2014	0.03	7.3 ± 0.10	279 ± 3.9	114.5 ± 3.9	1	**4.33± 0.02**	4.33	58.9	4.5–10.0	0.45–1000
	**K (SS336)**	Sta.1’	19 Aug, 2014	0.05	29.5 ± 0.18	580 ± 3.5	567.3 ± 3.5	1	**0.65 ± 0.01**	0.65	2.2		
	**L (SS386)**	Sta.1’	14 Oct, 2014	0.22	55.0 ± 1.7	248 ± 7.9	243.2± 7.9	1	**1.07 ± 0.01**	1.07	1.9		

^137^Cs activity concentration without CsMPs (Bq/g)

^a^: (total ^137^Cs in bulk sample (Bq)–total ^137^Cs in CsMPs (Bq)) / sample weight (g). For caddisfly larvae, the actual values of re-measurement by high purity germanium (HPGe) of samples after removal of CsMP are shown. ^137^Cs activity (Bq)

^b^: ^137^Cs in CsMPs were measured by autoradiography. The separated CsMPs were shown in bold and measured by HPGe for ^137^Cs activity.

### Radioactivity measurements of the bulk samples

The radioactivity of caddisfly and dobsonfly larval samples were measured using a well-type high-purity germanium coaxial detector due to their small quantity (Canberra GCW6023; Mirion Technologies, Canberra Ltd., Tokyo, Japan) in a flat-bottom test tube (diameter = 15 mm, height = 57 mm). Periphyton and CPOM samples were stored in plastic containers (U8 container, diameter = 50 mm, height = 62 mm), and ^137^Cs activity concentrations were measured using HPGe (Canberra GCW6023; Mirion Technologies, Canberra Ltd., Tokyo, Japan. and SEG-EMS GEM 30–70; SEIKO EG&G Ltd., Tokyo, Japan). SS samples on the wet cartridge filter were measured using HPGe (Canberra GC2518, Mirion Technologies, Canberra Ltd., Tokyo, Japan) in a plastic polyacetal case, and geometric corrections were determined the actual amount of ^137^Cs in the U8 container [20]. Gamma Studio software (SEIKO EG&G, Tokyo, Japan) and Spectrum Explorer (Mirion Technologies, Canberra Ltd., Tokyo, Japan) were used to analyze the γ-ray spectra. The counting errors were calculated by the software considering gross counts and background counts. Most samples were measured for <10% of counting error, except for several aquatic insect samples of small quantities in flat-bottomed test tubes that were measured for <15% of counting error. Standardized sources for calibrating the detectors were MX035 (Japan Radioisotope Association, Tokyo, Japan) for well-type germanium coaxial detectors and MX033U8PP (Japan Radioisotope Association, Tokyo, Japan) for the U8 container. The activities of the samples were corrected for radioactive decay to the date of sample collection. The supporting information include the ^137^Cs activity concentrations of the bulk samples of insects, periphyton, POM, and SS (S1 Data) and ^137^Cs activity concentrations of the individual insect samples at OD1 (S2 Data).

### Isolation and radioactivity measurement of radioactive particles

**Fig 2:**
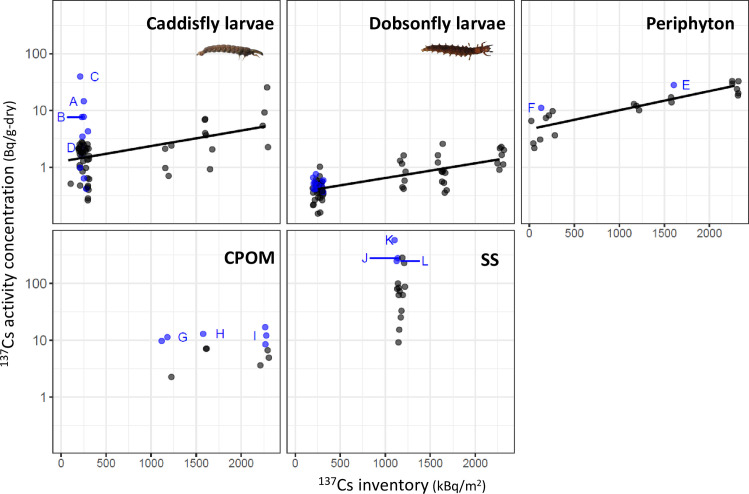
The ^137^Cs activity concentration in aquatic insects, periphyton, coarse particulate organic matter (CPOM), and suspended solids (SS) samples. The points are jittered in the x-axis to avoid overplotting. Blue dots indicate samples provided for autoradiography, and alphabets A to L indicate the samples where CsMPs were observed (see Table 2). The lines represent linear model prediction (S1 Table). ^137^Cs activity concentration of SS have been reported in Tsuji et al. [19].

After radioactivity measurement, total ^137^Cs activity of caddisfly and dobsonfly larvae samples were calculated, and autoradiography analysis was performed on the samples with the ten highest total ^137^Cs activity levels (excluding samples that were not present because they were used for other analyses) (S1 Fig). In addition, two samples of periphyton, six samples of CPOM, and three samples of SS with relatively high ^137^Cs activity concentrations were used (Fig 2, blue points).

The spatial distribution of radioactivity in the samples was measured by autoradiography using imaging plate (IP, BAS-MS 2040; Fujifilm Ltd., Tokyo, Japan) and an IP reader (FLA-9000; Fujifilm Ltd., Tokyo, Japan). The IP was then applied to samples for 30 min. Three CsMPs with known ^137^Cs activity (~1 Bq, 3 Bq, and ~12 Bq) along with the samples were in contact with the IP as calibration standards for calculating ^137^Cs at each hot spot (S2 and S3 Figs). Radioactive particles (RPs) in the samples were identified from the images obtained using an IP reader.

In the current study, RPs with more than 0.1 Bq of ^137^Cs calculated using IP images were considered as CsMPs based on the study of Miura et al. [18]. RPs with relatively high radioactivity were isolated using the wet separation method [15, 18], which enabled the efficient isolation of RPs. The isolated RPs were identified using scanning electron microscopy (SEM; TM3030Plus, Hitachi Ltd., Tokyo, Japan) with energy dispersive X-ray spectroscopy (EDS; AZtecOne, Oxford Instruments Ltd., UK). Subsequently, ^134^Cs (604.7 keV) and ^137^Cs (661.7 keV) activities in the isolated RPs were determined by gamma-ray spectrometry using an HPGe (GX4018; Mirion Technologies, Canberra Ltd. Tokyo, Japan) to determine the unit of FDNPP from which the RPs originated. Radioactivity standard solutions for ^134^Cs (0.182 Bq as of November 25, 2016, Japan Radioisotope Association, CZ-010) and ^137^Cs (1.40 Bq as of November 25, 2016, Japan Radioisotope Association, CS-005) dispersed on a filter of 1 mm square were used for calibration of the gamma-ray spectrometer. The RPs and the standard filter which had similar RCs activity to that of CsMPs were measured at the same position from the detector. The activities of RPs were calculated by counting efficiency of the sample and the standard filter. In addition, relative efficiency of HPGe was 23%. Therefore, no correction for the sum effect of ^134^Cs was needed in this study. The radioactivity standard solutions were calibrated using the Japan Calibration Service System (JCSS; http://www.nite.go.jp/en/iajapan/jcss/index.html). The activities of RPs were corrected to the date of the sample collection and to that of the accident for the calculation of ^134^Cs/^137^Cs activity ratio.

### Calculation of radioactivity fraction and frequency of CsMPs

To investigate the contribution of CsMPs to the total ^137^Cs, the radioactivity fractions of CsMPs were calculated for caddisfly larvae, periphyton, CPOM, and SS samples as follows:

$$Radioactivity\;fraction\;of\;CsMPs\;(\%)=\frac{Total\;{}^{137}Cs\;activity\;in\;CsMPs}{Total\;{}^{137}Cs\;activity\;in\;bulk\;sample}\times 100$$

The total ^137^Cs in CsMPs (Bq) was calculated as the sum of all ^137^Cs in CsMPs quantified by IP image or HPGe, and that in bulk samples was calculated from the ^137^Cs activity concentration quantified by the well-type HPGe. The activity level of each sample was decay-corrected to the sampling date. Due to the large radioactivity fraction of CsMPs in caddisfly samples, the total ^137^Cs in CsMPs can be greater than that of the bulk sample by HPGe, which resulted in fractions exceeding 100% (Table 2B). Therefore, the fractions for the caddisfly sample (A–D) as

$$Radioactivity\;fraction\;of\;CsMPs\;(\%)=\frac{Total\;{}^{137}Cs\;in\;CsMPs}{\left(Total\;{}^{137}Cs\;in\;CsMPs)+\;(Total\;re–measured\;{}^{137}Cs\;in\;bulk\;sample\;without\;CsMPs\right)}\times 100$$

The frequency of CsMPs was calculated by dividing the number of CsMPs by sample weight (Table 2). The frequency of CsMPs of periphyton, CPOM, and SS was calculated for both the samples provided for autoradiography (blue points in Fig 2; the calculated frequency is an upper limit and is likely to be somewhat overestimated) and all collected samples (all points in Fig 2, the calculated frequency is a lower limit and somewhat underestimated). For caddisfly larvae, the frequency of CsMPs was calculated for digestive tract content. In our preliminary experiment, the ethanol-preserved caddisfly larvae, excluding those used in this experiment, were dissected using scissors, and each body part (digestive tract with its content, muscle tissues, and head and skin tissues) of about 10 individuals was pooled together, dried, and weighed. Because the digestive tract weight was approximately 10%–30% of the whole body weight, we calculated the digestive tract weight as 10%–30% of the total weight of 46 individuals.

### Statistical analysis

To examine the relationship between inventory and ^137^Cs activity concentrations in aquatic insects, attached algae, and CPOM, we conducted a linear regression analysis using the log-transformed ^137^Cs activity concentrations as objective variables and inventory values of sampling sites as explanatory variables. For aquatic insects, the species (caddisfly larvae or dobsonfly larvae) was added as an explanatory variable to investigate the differences in ^137^Cs activity concentration among species. In addition, the relationship between ^137^Cs activity concentration and the size of aquatic insects was analyzed by linear regression analysis using the log-transformed ^137^Cs activity concentrations as objective variables and the weight of individuals as explanatory variables. All statistical analyses were conducted using R ver. 4.1.1, a programming language for statistical computing and graphics supported by the R Core Team and the R Foundation for Statistical Computing [21].

## Results and discussion

### ^137^Cs activity concentration of aquatic insects, periphyton, CPOM and SS

The activity concentrations of ^137^Cs in the collected samples in each medium varied from one order of magnitude (dobsonfly larvae, periphyton, and CPOM) to two orders of magnitude (caddisfly larvae and SS) at the same sampling site (Fig 2). The ^137^Cs activity of aquatic insects and periphyton were positively correlated to ^137^Cs inventory, and ^137^Cs activity concentration was higher in the upstream area than that in the downstream area (Fig 2; regression results are listed in S1 Table).

For aquatic insects, the variability of ^137^Cs activity concentration in each individual caddisfly larvae at OD1 was significantly larger (coefficient of variation, CV = 2.06) than that of bulk samples at other sites (see Fig 2; CV at Sta.1 = 0.54, CV at Sta.2 = 0.61, CV at Sta.3 = 0.97). Aquatic insects were revealed to have a largely varied ^137^Cs activity concentration between individuals, which was not apparent when measured as a bulk sample. The ^137^Cs activity concentrations of caddisfly larvae were significantly higher than those of dobsonfly larvae (S1 Table, regression results of the aquatic insects model). This result was consistent with a previous study showing that detritivorous aquatic insects have higher ^137^Cs activity concentrations than that in carnivores [4].

**Fig 3:**
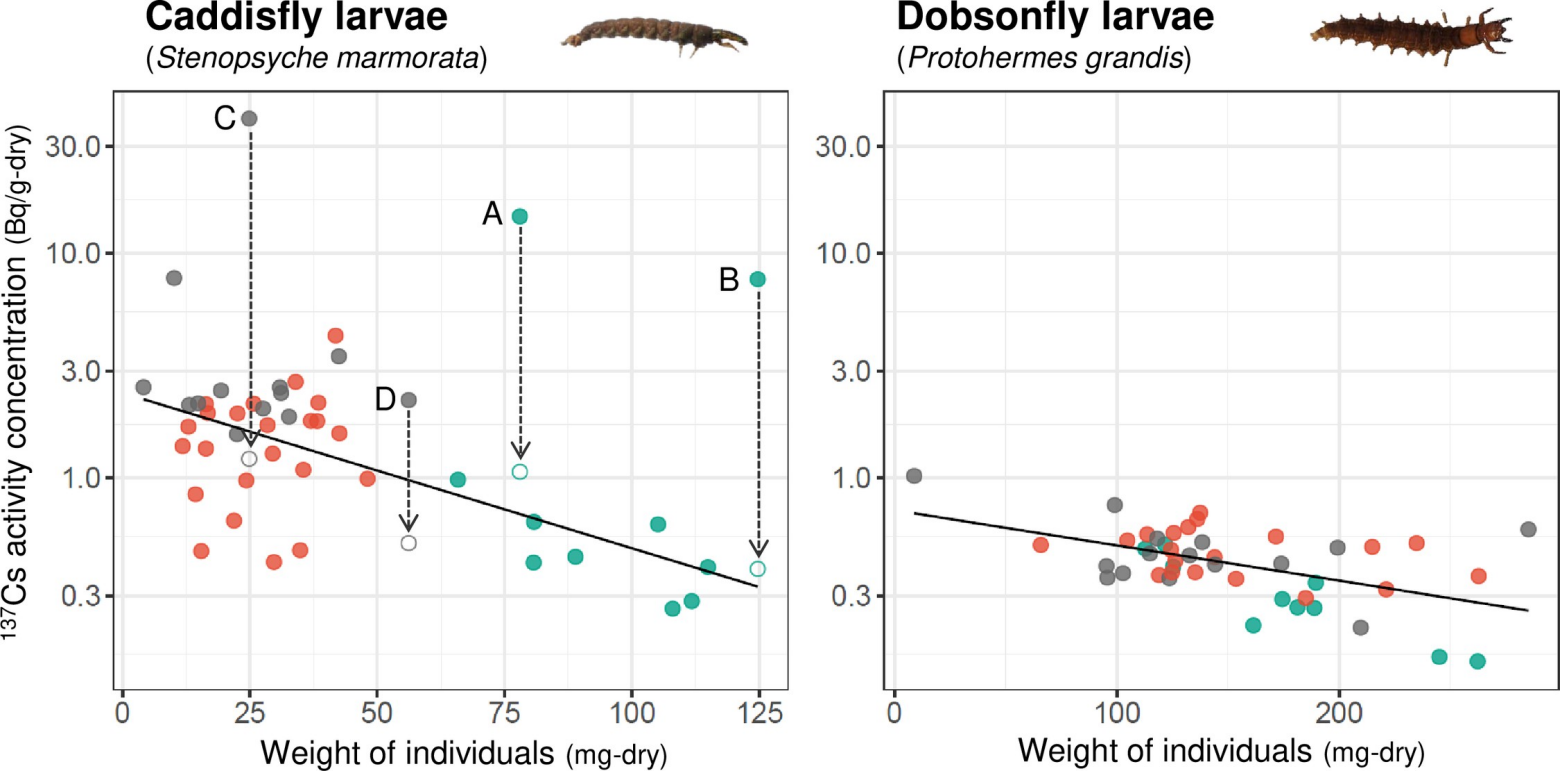
Relationship between ^137^Cs activity concentration and weight of individuals in aquatic insects. Aquatic insects were collected at OD1 in summer (green), autumn (gray), and winter (red). Alphabets A to D indicate the sample ID where CsMPs were observed (see Table 2). White points indicate ^137^Cs activity concentration from the samples after removing CsMPs. The lines represent linear model prediction (S2 Table).

Fig 3 shows the ^137^Cs activity concentration of aquatic insects against their body weight at OD1. The ^137^Cs activity concentration in caddisfly and dobsonfly larvae was inversely proportional to body size. Among the caddisfly larvae, three samples showed relatively high ^137^Cs activity concentrations (A–C). Autoradiography analysis of ten samples with the highest total ^137^Cs radioactivity found RPs with ^137^Cs > 0.1 Bq from four samples (A–D), and ^137^Cs activity concentration dropped significantly after the RPs were removed (Fig 3, described in detail in the next section). When the values of A–D were replaced with ^137^Cs activity concentration without RPs, the regression model revealed a significant negative effect on body weight in both caddisfly larvae and dobsonfly larvae (S2 Table). The body size of caddisfly larvae differed among seasons depending on their developmental stage (Fig 3). At OD1, body sizes of caddisfly larvae were larger in summer and smaller in autumn and winter, probably due to adult hatching and emergence of wintering generations after summer (August). Caddisfly larvae have one or two generations per year, whereas dobsonfly larvae develop for several years in the water; therefore, dobsonfly larvae of various sizes exist regardless of the season. The effect of body size on ^137^Cs activity concentration may be explained by the fact that ^137^Cs activity concentration of the digestive tract content is much higher than that of the rest of the body in caddisfly larvae [5], and the ratio of the weight of the digestive tract content to total body weight is smaller in larger individuals (Ishii and JO, unpublished). Our preliminary dissection experiment described in the Materials and methods revealed that the ratio of digestive content weight of caddisfly larvae is 10–30% of the whole body weight, with larger individuals tending to have smaller ratios. Additionally, in dobsonfly larvae, the ^137^Cs activity measurement of each dissected body part showed that ^137^Cs activity concentration of the digestive tract was also higher than that in the rest of the body (Ishii and JO, unpublished).

### Characterization of CsMPs

**Fig 4:**
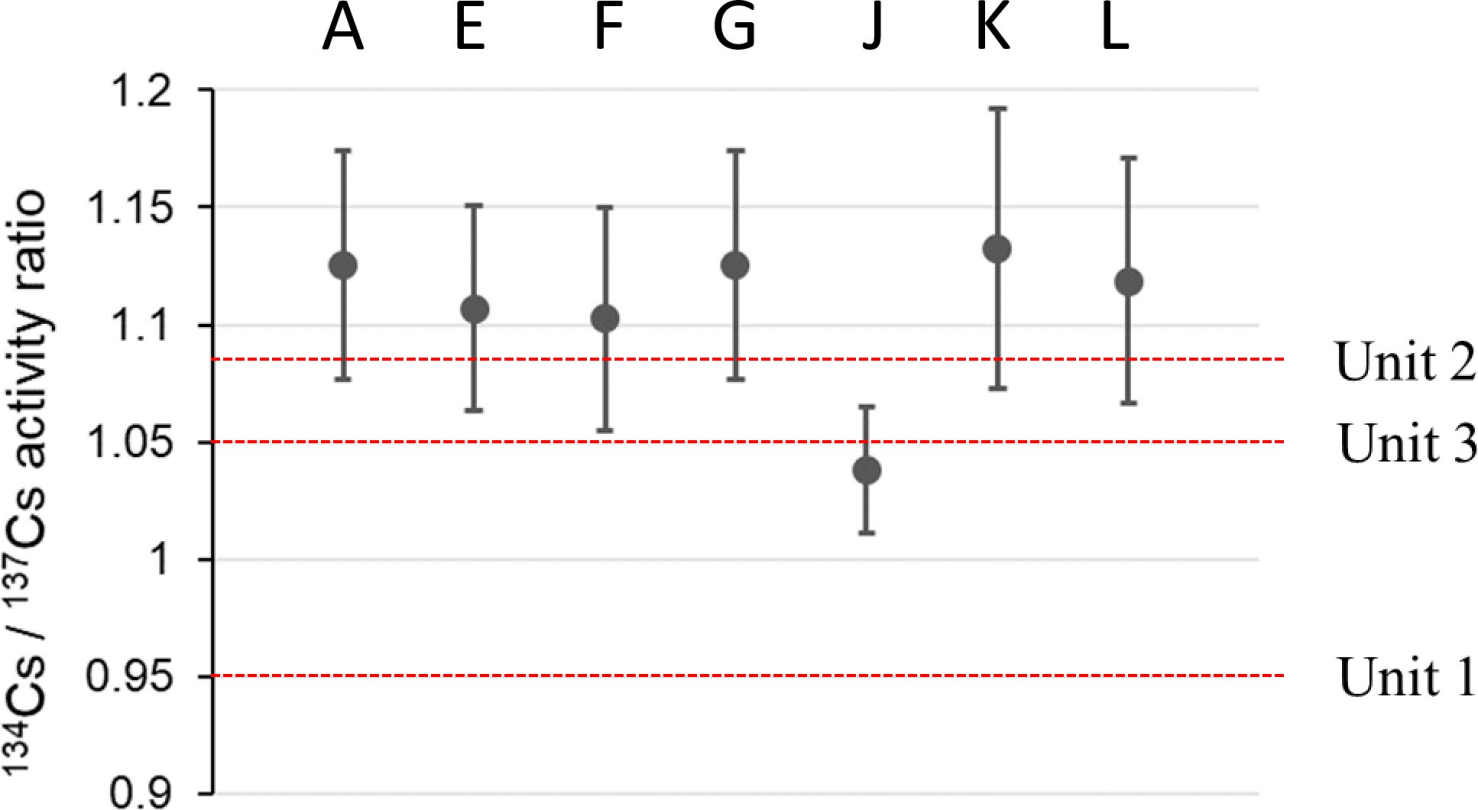
Cesium activity ratios (^134^Cs / ^137^Cs) of radiocesium-bearing microparticles (CsMPs). The letters A to L indicate the ID of the CsMPs (Table 2). The values of the damaged reactor cores (Units 1–3) are cited from Nishihara et al. [22].

**Fig 5:**
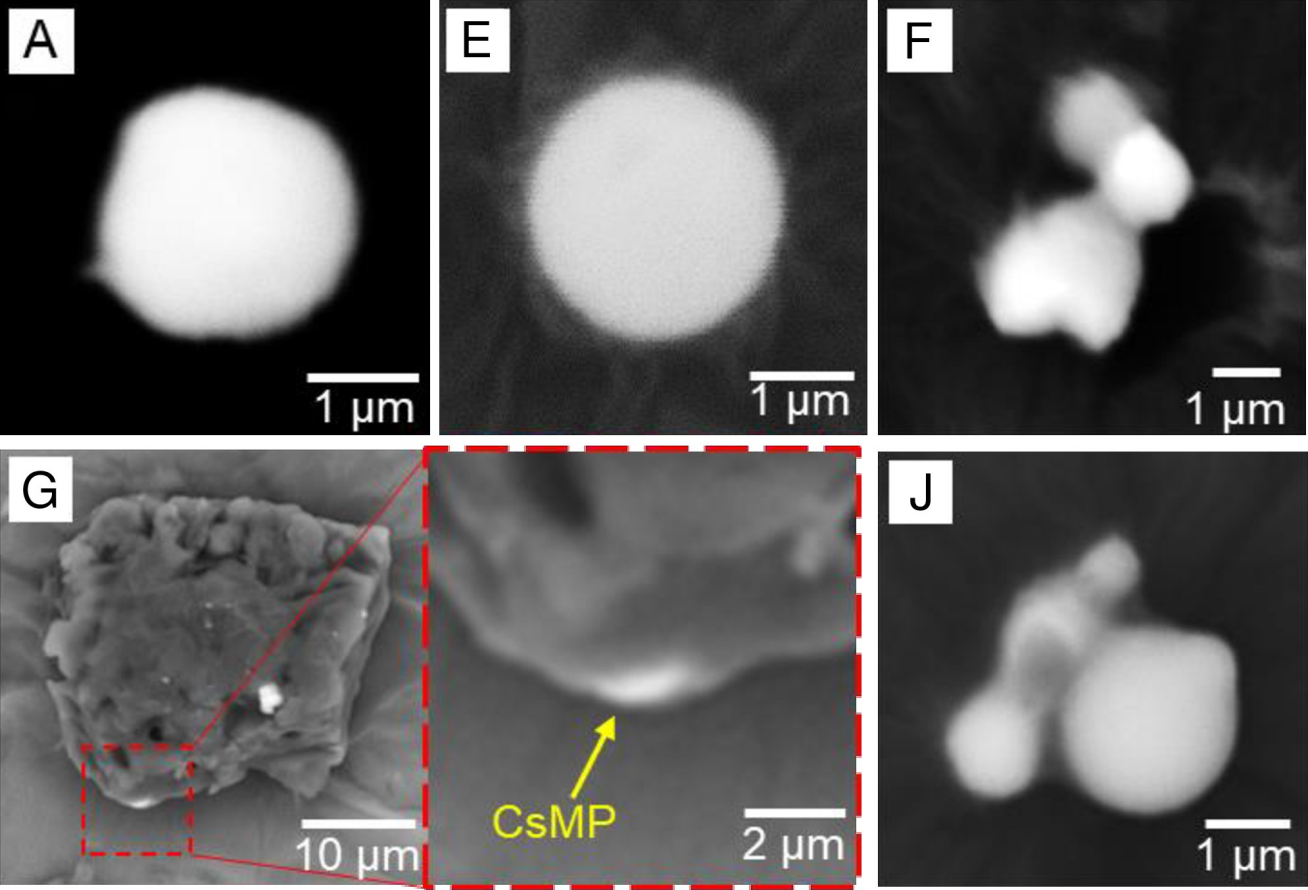
Scanning electron microscope (SEM) images of radiocesium-bearing microparticles (CsMPs). CsMPs were isolated from caddisfly larvae (A), periphyton (E, F), coarse particulate organic matter (CPOM) (G) and suspended solids (SS) (J). CsMPs were placed on Kapton tapes for SEM-EDS analyses. For the CsMP from G, Cs was detected only in the white area of the large particle.

**Fig 6:**
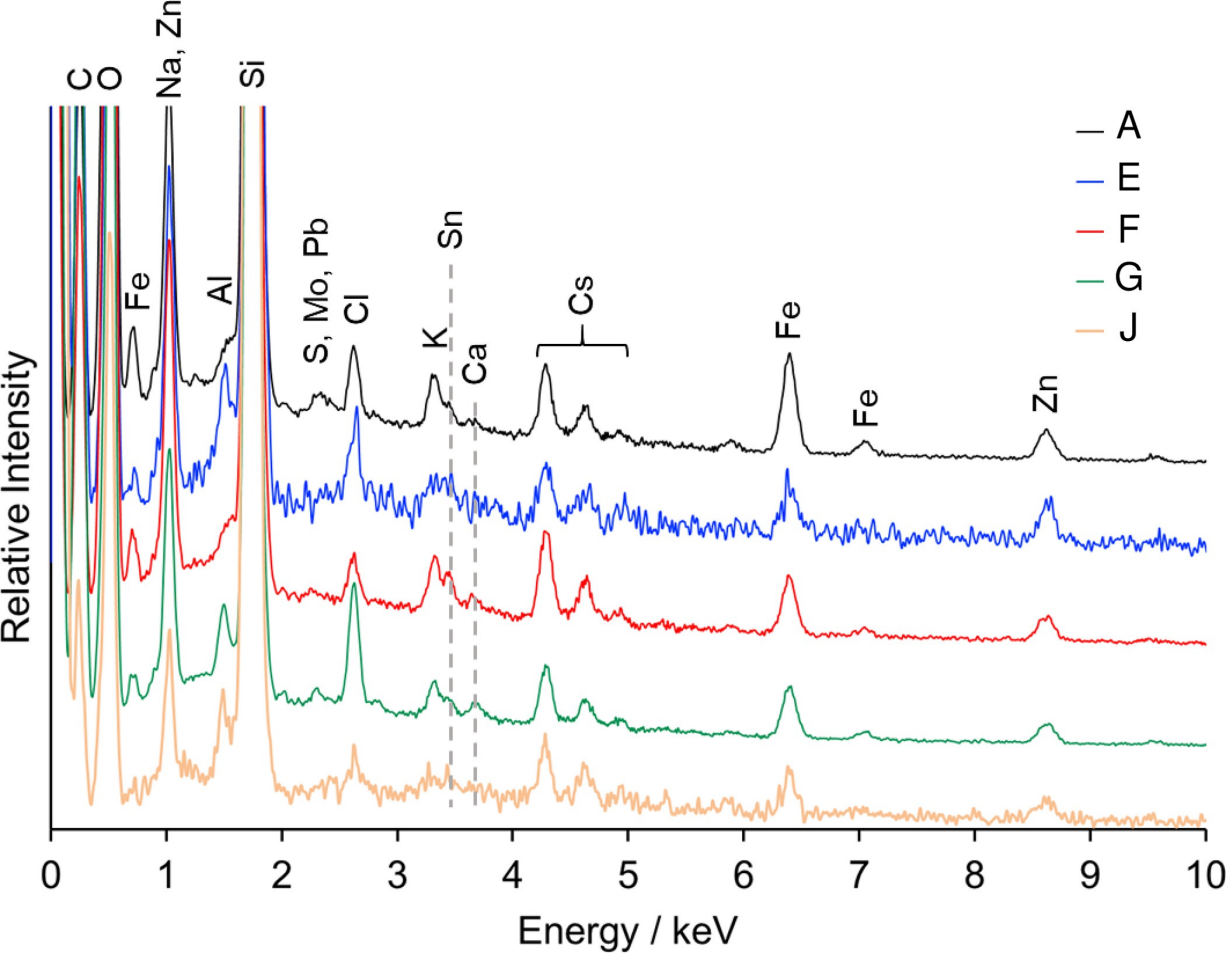
Energy-dispersive X-ray (EDS) spectra of radiocesium-bearing microparticles (CsMPs). Carbon was obtained from carbon coating.

We used autoradiography to identify and isolate RPs from aquatic insects and their diet sources, periphyton, CPOM, and SS samples. The ^137^Cs radioactivity of RPs were quantified by IP images, and RPs with more than 0.1 Bq were assumed to be CsMPs. Standard deviation of the radioactivity measurement by IP image was less than 10%. Table 2 lists the total number of CsMPs found in each sample. Seven CsMPs (A, E, F, G, J, K, and L in Table 2) with relatively high ^137^Cs were isolated from the bulk samples, and the ^134^Cs and ^137^Cs radioactivities were measured by HPGe. The ^134^Cs/^137^Cs activity ratio of each CsMP (Fig 4) suggested that these CsMPs were emitted from Unit 2 or 3 of the FDNPP because the ratios of each CsMP were around 1.08 (Unit 2) and 1.05 (Unit 3), as calculated by Nishihara et al. [22]. Among the isolated CsMPs, five (A, E, F, G, and J) were analyzed by SEM-EDS measurements (Figs 5 and 6). The CsMPs were a few microns in size (Fig 5). The results of the EDS analysis showed peaks of Si, Cl, K, Fe, Zn, and Sn with Cs (Fig 6). The Al and Ca peaks were possibly due to secondary adhesion [18]. These isolated CsMPs were considered to be Type-A particles based on the ^137^Cs radioactivity, ^134^Cs/^137^Cs activity ratio, size, and elemental composition. The CsMPs from CPOM (G) were attached to a large particle (~30 μm; Fig 5G; S4 Fig). Miura et al. [10] also reported Type-A particles attached to large particles in marine samples. The presence of Type-A particles in river SS has been reported in the Kuchibuto River [18]; however, to the best of our knowledge, the current study documents Type-A particles in aquatic insects and periphyton for the first time. According to Ikehara et al. [23], Type-A particles were transported by plume 2 (period: from night of 14 to morning of March 15, 2011) and plume 3 (afternoon of March 15, 2011), as reported by Tsuruta et al. [24]. In particular, plume 3 passed over the Ota River basin [25], which suggests that CsMPs originating from the basin can be present in the Ota River.

### The effect of CsMPs on ^137^Cs variability of aquatic insects

CsMPs were found in four caddisfly samples (Fig 3; Table 2A–2D), three of which had high ^137^Cs activity concentrations. To confirm the influence of CsMPs on the ^137^Cs activity concentration of caddisfly larvae, CsMPs were removed from the samples during wet separation, and the remaining were re-measured for ^137^Cs activity using HPGe. ^137^Cs activity concentrations decreased to the same level as that of the other caddisfly larvae (Fig 3). This result confirmed that the presence of CsMPs caused sporadic high ^137^Cs activity concentration in caddisfly larvae, especially when the ^137^Cs activity in CsMPs was high (Table 2A–2C) compared to when the ^137^Cs activity in CsMPs was low (Table 2D). No CsMPs were found and no samples with extremely high ^137^Cs activity concentrations were found in dobsonfly larvae samples when 45 individuals were investigated, compared to 4 out of 46 in caddisfly larvae. Although autoradiography was performed on only the samples with the ten highest total ^137^Cs radioactivity values, CsMP of > 0.1 Bq was unlikely to be found in other samples of both sample groups, given that total ^137^Cs radioactivity of these bulk samples was less than 0.1 Bq (S1 Fig). The higher probability of CsMPs being found in caddisfly larvae than that in dobsonfly larvae (*P* = 0.04, one-sided z-test) may be attributed to their different feeding habits. In contrast to carnivorous dobsonfly larvae, detritivorous caddisfly larvae feed on drifting fine particulate organic matter captured in nets and hence are more likely to take up CsMPs with dietary particulates [2]. The CsMPs were likely in their digestive tract because (i) CsMPs were found in caddisfly larvae, which are more likely to consume CsMPs; no CsMPs were found in dobsonfly larvae, and (ii) CsMPs on the body surface of aquatic insects are likely to have been removed when samples were preserved in ethanol after collection.

After the removal of the CsMPs, the inter-individual variability in ^137^Cs activity concentrations of caddisfly larvae was still greater than that of dobsonfly larvae (CV of caddisfly larvae without CsMPs = 0.77, CV of dobsonfly larvae = 0.36). Differences in the amount of gut contents between individuals will have a large effect on whole-body ^137^Cs radioactivity due to the high ^137^Cs activity of gut content in caddisfly larvae. In addition, although RPs above > 0.1 Bq were considered as CsMPs in this study, CsMPs with ^137^Cs radioactivity less than 0.1 Bq and weathered biotite with ^137^Cs radioactivity more than 0.1 Bq have been reported [26]. As the RPs with < 0.1 Bq radioactivity were actually observed in the autoradiography of caddisflies, these RPs in the caddisfly diet may also affect the variability of ^137^Cs activity concentration.

### Radioactivity fraction of CsMPs

The radioactivity fractions of CsMPs differed between caddisfly larvae, periphyton, CPOM, and SS samples (Table 2). The radioactivity fraction of the CsMPs was high (77%–96%) in caddisfly larvae and relatively low in periphyton (< 20%), CPOM (< 10%), and SS samples (58% in sample J and about 2% in the other two samples). The CsMP radioactivity fraction has an inverse correlation with the total radioactivity in the bulk sample. Briefly, the effect of a single CsMP inclusion on CsMP fraction is larger if the total ^137^Cs radioactivity of the bulk is low, and is smaller if the total ^137^Cs radioactivity is high. As the total radioactivity of the caddisfly bulk sample without CsMPs was small (< 0.2 Bq) because of its relatively low ^137^Cs activity concentration and small sample weight (Table 2), the CsMP radioactivity fraction was large, and CsMP incorporation resulted in a sporadically high ^137^Cs activity concentration. The total radioactivity of other bulk samples was larger in periphyton (17–220 Bq), CPOM (47–285 Bq), and SS (3–54 Bq) than that of caddisfly due to the larger sample weight for periphyton and CPOM samples, and the higher ^137^Cs activity concentration in SS samples, resulting in a small CsMP radioactivity fraction and a small effect of CsMPs on ^137^Cs activity concentration. Thus, it should be noted that the inclusion of CsMPs could substantially increase the measured value of ^137^Cs activity concentration, especially for samples with low total radioactivity, such as small insects or plankton samples [10].

### The frequency of CsMPs in caddisfly and their diet

We investigated the frequency of CsMPs in caddisfly larvae and their dietary sources to determine the intake route of CsMPs by the larvae. The frequency of CsMPs in the caddisfly digestive tract content (5.3–15.9) was close to that of CsMPs in SS (4.5–10.0) (Table 2). This result was reasonable because caddisfly larvae feed on fine particles classified as fine particulate organic matter (FPOM) (0.45–1,000 μm), whose particle size is almost consistent with that of SS (0.45–1,000 μm) [20]. As CsMPs (0.1–10 μm) are also included in FPOMs, caddisflies are likely to take up CsMPs with other suspended particles, such as drifting periphyton and phytoplankton, which are captured in nets of these larvae [5, 27].

The frequency of CsMPs was much lower for periphyton (0.1–0.8) and CPOM (0.3–0.5) samples than that for caddisfly larvae digestive tract content. This was not surprising because the particle size of the CPOM was > 1,000 μm, and most CsMPs were sieved off during pretreatment. CsMPs found in the CPOM were mostly likely attached to large organic particles, as shown in Fig 5G. Additionally, CsMP frequency in periphyton was low; however the frequency was difficult to interpret. Although the particle size of periphyton tissue itself was approximately 2–200 μm [28], the collected sample contained particles of all sizes, including SS and CPOM, accumulating within a periphyton matrix. The frequency of CsMPs in periphyton can be diluted by these accumulated particles. These results suggest that caddisfly larvae selectively feed on FPOM (SS in this study) similar in size to CsMPs from drifting organic matter and consequently collect CsMPs efficiently.

### Uptake of CsMPs into aquatic ecosystems

Our study revealed that CsMPs were distributed throughout the environment in the Ota River, including drifting particulates, periphyton, and aquatic insects in river water. CsMPs are suggested to be deposited throughout a wide area of eastern Japan, for example in Fukushima Prefecture and the adjacent Ibaraki Prefecture [11, 12, 18]. Therefore, their uptake by organisms might occur commonly in the areas deposited with CsMPs. This consequently raises concerns about the mechanism by which CsMPs affect the radiological risk of aquatic organisms and indirectly humans. For caddisfly larvae, the gut content is expelled from the digestive tract within a few days [5]; thus, CsMPs are also likely to be excreted from the digestive tract with other contents. Furthermore, the bioavailability of RCs in CsMPs is low. Although CsMPs are reported to eventually dissolve in water, Type-A particles (1 μm) may take decades to completely dissolve in pure water (pH = 5, 13°C) [29]. Therefore, even if CsMPs are taken up by fishes or humans through diet, the dissolution rate of bioavailable RCs can be very small before CsMPs are expelled from the digestive tract. However, because pH and temperature affect the dissolution rate of CsMPs [29], and further studies on the dissolution of CsMPs in the digestive tract are required.

### Conclusion and future work

The results of this study suggested that CsMPs could be taken up by aquatic organisms. In caddisfly larvae, the activity concentration of ^137^Cs varied greatly among individuals, and CsMP uptake was responsible for sporadically high RCs concentrations. Radioactivity measurement of aquatic insects with small numbers of individuals, rather than tens of them, reduced collection effort and revealed variations in RCs concentrations between individuals, improving our understanding of RCs transfer. However, considering the incorporation of CsMPs is crucial because this may cause sporadically high RCs concentrations and overestimation of RCs concentrations in aquatic insects. This study did not investigate the uptake of CsMPs by fish through the consumption of aquatic insects. Since CsMPs may be taken up by organisms in both aquatic and terrestrial ecosystems via food webs, these uptakes and their impact on RCs concentration measurements need to be further studied in future research.

## Supporting information

S1 Fig Total ^137^Cs in the aquatic insect samples. (DOCX)

S2 Fig Calibration of photo stimulated luminescence (PSL) by ^137^Cs radioactivity in CsMPs. (DOCX)

S3 Fig The autoradiography observation of the CsMPs for the aquatic insect samples. (DOCX)

S4 Fig Energy-dispersive X-ray spectra of Particle X and matrix of sample G. (DOCX)

S1 Table Results of the linear regression analysis of the inventory and the ^137^Cs activity concentrations in aquatic insects, periphyton, and coarse particulate organic matter (CPOM). (DOCX)

S2 Table Results of the linear regression analysis of ^137^Cs activity concentration and weight of individuals in aquatic insects. (DOCX)

S1 Data ^137^Cs activity concentrations of the bulk samples of insect, periphyton, POM and SS samples. (XLSX)

S2 Data ^137^Cs activity concentrations of individual insect samples at OD1. (XLSX)
