# Supplementary material for: Radiocesium-bearing microparticles cause a large variation in 137Cs activity concentration in the aquatic insect Stenopsyche marmorata (Tricoptera: Stenopsychidae) in the Ota River, Fukushima, Japan
Source: PLoS One. 2022 May 20;17(5):e0268629. doi: 10.1371/journal.pone.0268629 (PMC9122184; doi:10.1371/journal.pone.0268629)
Supplement: S1 Fig — (DOCX) [file pone.0268629.s001.docx]

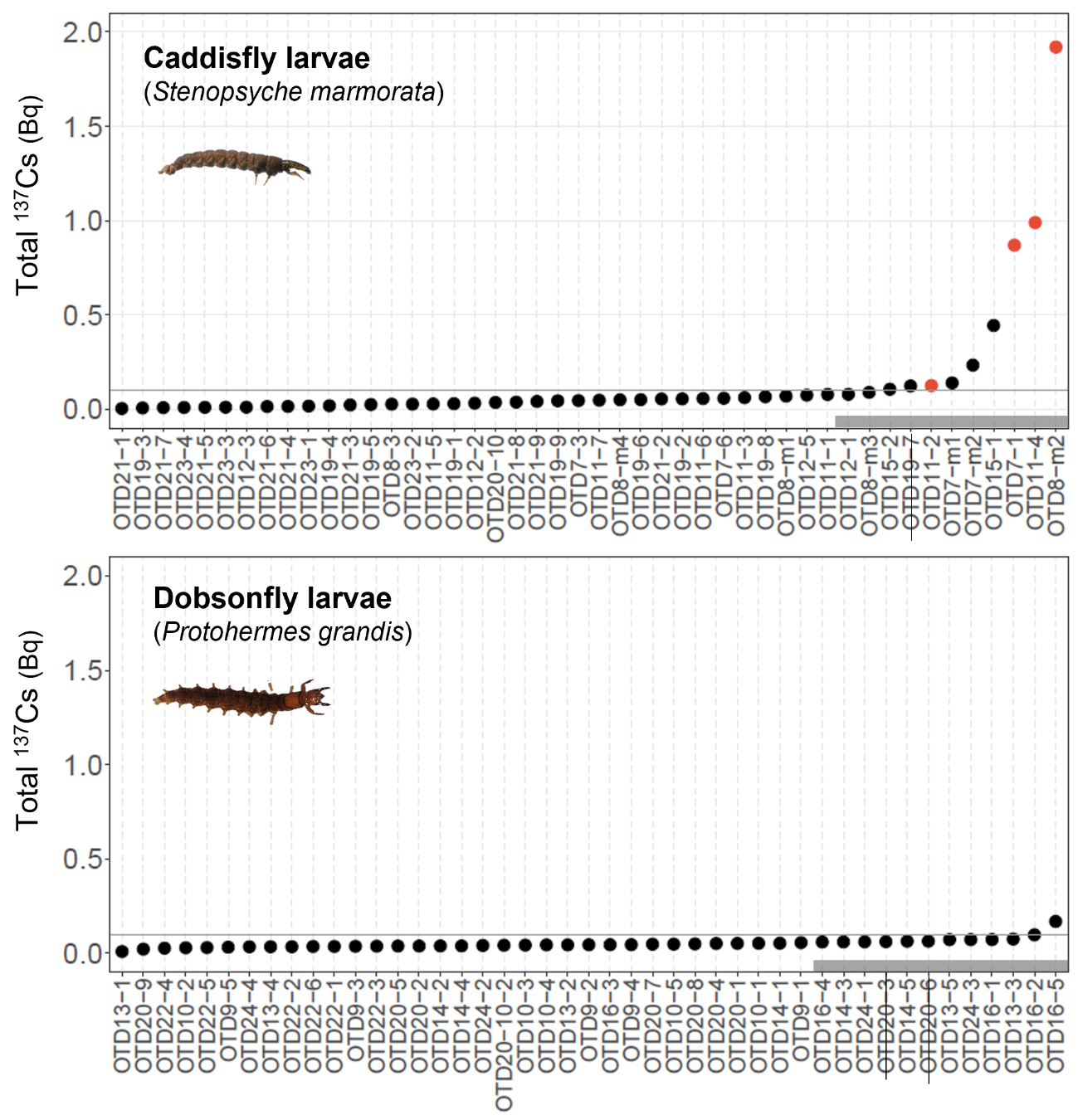


**S1 Fig.** Total ^137^Cs in the aquatic insect samples. The total ^137^Cs is shown in an increasing order. Horizontal line indicates 0.1 Bq. Autoradiography analysis was performed on the samples shown in the bold gray horizontal line from the right end (top 10 highest total ^137^Cs activities excluding strike-out samples used for other analyses). Red dots indicate samples with radioactive particles with ^137^Cs activity > 0.1.
