# Supplementary material for: Radiocesium-bearing microparticles cause a large variation in 137Cs activity concentration in the aquatic insect Stenopsyche marmorata (Tricoptera: Stenopsychidae) in the Ota River, Fukushima, Japan
Source: PLoS One. 2022 May 20;17(5):e0268629. doi: 10.1371/journal.pone.0268629 (PMC9122184; doi:10.1371/journal.pone.0268629)
Supplement: S2 Fig — (DOCX) [file pone.0268629.s002.docx]

**
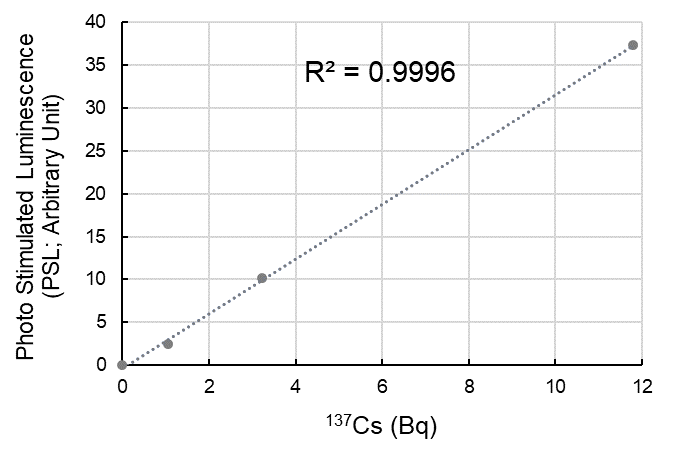
**

**S2 Fig.** Calbration of photo stimulated luminescence (PSL) by ^137^Cs radioactivity in CsMPs. The radioactivity of three standard CsMPs are about 1 Bq, 3 Bq and 12 Bq.
