# Supplementary material for: Radiocesium-bearing microparticles cause a large variation in 137Cs activity concentration in the aquatic insect Stenopsyche marmorata (Tricoptera: Stenopsychidae) in the Ota River, Fukushima, Japan
Source: PLoS One. 2022 May 20;17(5):e0268629. doi: 10.1371/journal.pone.0268629 (PMC9122184; doi:10.1371/journal.pone.0268629)
Supplement: S3 Fig — (DOCX) [file pone.0268629.s003.docx]

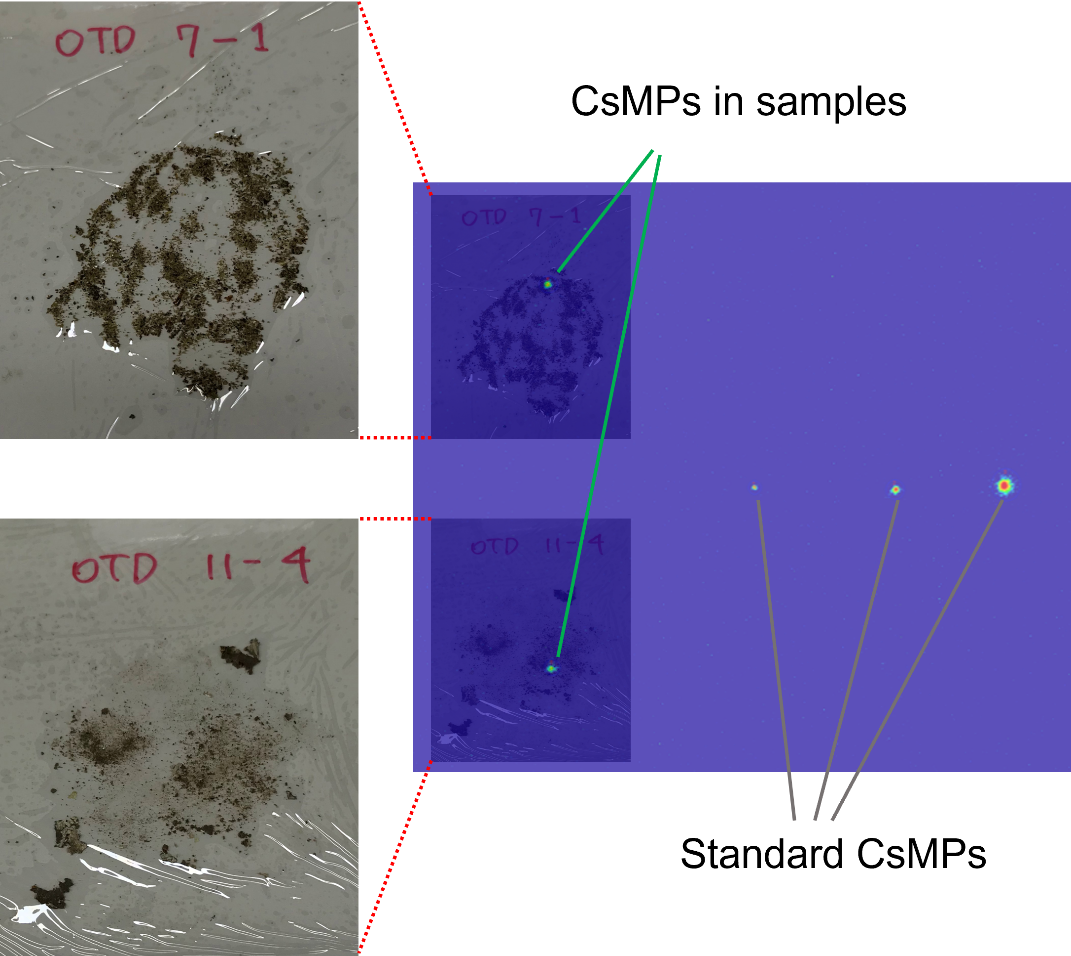


**S3 Fig.** The autoradiography observation of the CsMPs for the aquatic insect samples. Two wrapped samples (OTD 7-1, OTD 11-4) and the three standard CsMPs (~1 Bq, 3Bq, 12 Bq) were measured at the same time.
