# Supplementary figures and images for: Radiocesium-bearing microparticles cause a large variation in 137Cs activity concentration in the aquatic insect Stenopsyche marmorata (Tricoptera: Stenopsychidae) in the Ota River, Fukushima, Japan
Source: PLoS One. 2022 May 20;17(5):e0268629. doi: 10.1371/journal.pone.0268629 (PMC9122184; doi:10.1371/journal.pone.0268629)

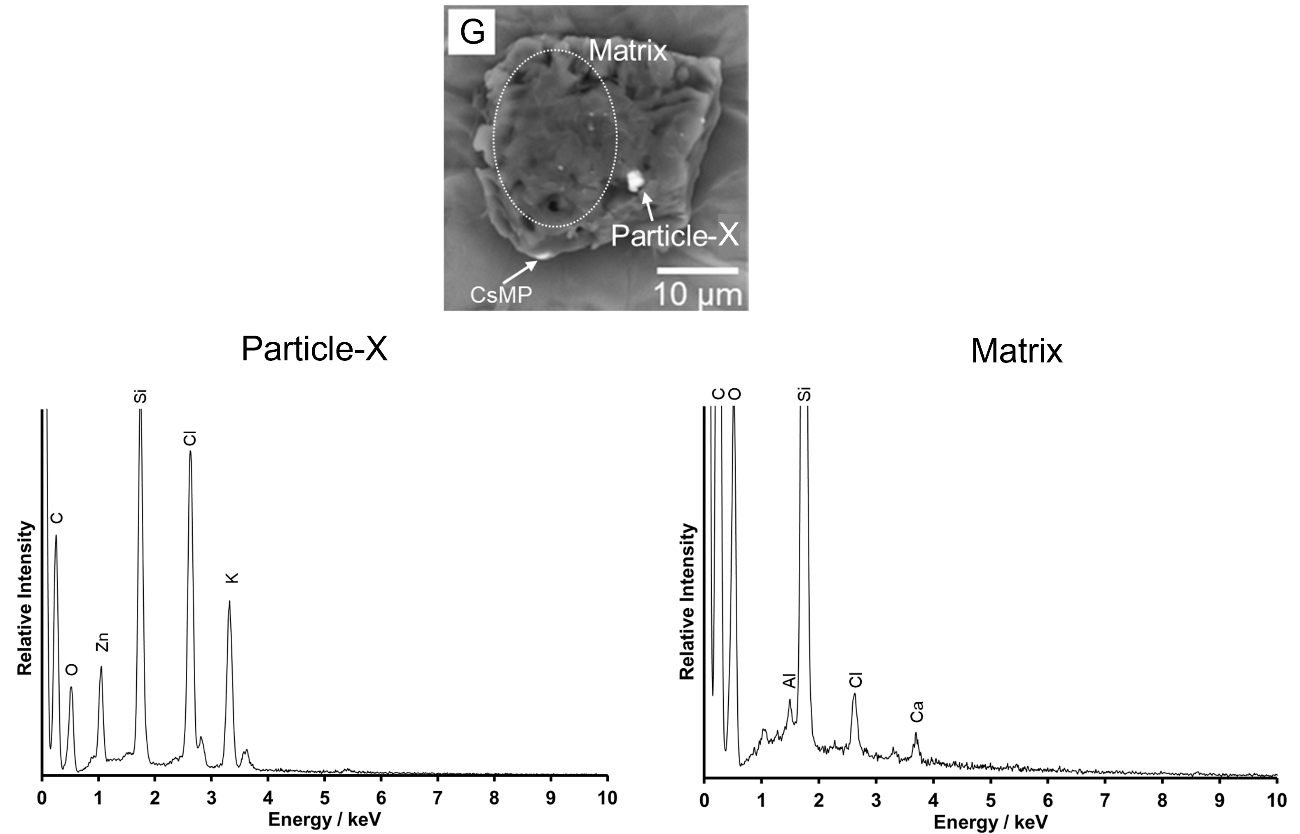


**S4 Fig.** Energy-dispersive X-ray spectra of the Particle-X and matrix of sample G.

Supplement: S4 Fig — (DOCX) [file pone.0268629.s004.docx]
