# Supplementary material for: Radiocesium-bearing microparticles cause a large variation in 137Cs activity concentration in the aquatic insect Stenopsyche marmorata (Tricoptera: Stenopsychidae) in the Ota River, Fukushima, Japan
Source: PLoS One. 2022 May 20;17(5):e0268629. doi: 10.1371/journal.pone.0268629 (PMC9122184; doi:10.1371/journal.pone.0268629)
Supplement: S1 Table — (DOCX) [file pone.0268629.s005.docx]

**S1 Table.** Results of the linear regression analysis of the inventory and the ^137^Cs activity concentrations in aquatic insects, periphyton, and coarse particulate organic matter (CPOM).

| **Model** | **Variable** | **Estimate** | **(CI)** | **P** |
| --- | --- | --- | --- | --- |
| Aquatic insects | Intercept | 6.70 | (6.81, 7.18) | **< 0.001** |
|  | Inventory | 0.0007 | (0.0005, 0.0008) | **< 0.001** |
|  | Species  (Dobsonfly larvae – Caddisfly larvae) | –1.61 | (–1.38, –0.94) | **< 0.001** |
| Periphyton | Intercept | 1.54 | (1.23, 1.85) | **< 0.001** |
|  | Inventory | 0.0008 | (0.0006, 0.0009) | **< 0.001** |
| CPOM | Intercept | 1.78 | (0.40, 3.15) | **0.02** |
|  | Inventory | 0.0001 | (–0.0006, 0.0009) | 0.73 |
