# Supplementary material for: Radiocesium-bearing microparticles cause a large variation in 137Cs activity concentration in the aquatic insect Stenopsyche marmorata (Tricoptera: Stenopsychidae) in the Ota River, Fukushima, Japan
Source: PLoS One. 2022 May 20;17(5):e0268629. doi: 10.1371/journal.pone.0268629 (PMC9122184; doi:10.1371/journal.pone.0268629)
Supplement: S2 Table — (DOCX) [file pone.0268629.s006.docx]

**S2 Table**. Results of linear regression analysis of ^137^Cs activity concentration and weight of individuals in aquatic insects

| **Model** | **Variable** | **Estimate** | **(CI)** | **P** |
| --- | --- | --- | --- | --- |
| Caddisfly larvae | Intercept | 0.87 | (0.59, 1.16) | **< 0.001** |
|  | Inventory | –0.016 | (–0.021, –0.010) | **< 0.001** |
| Dobsonfly larvae | Intercept | –0.33 | (–0.61, –0.04) | **0.03** |
|  | Inventory | –0.004 | (–0.005, –0.002) | **< 0.001** |
